# Development and Evaluation of Two Simple, Rapid Immunochromatographic Tests for the Detection of *Yersinia pestis* Antibodies in Humans and Reservoirs

**DOI:** 10.1371/journal.pntd.0000421

**Published:** 2009-04-28

**Authors:** Minoarisoa Rajerison, Sylvie Dartevelle, Lalao A. Ralafiarisoa, Idir Bitam, Dinh Thi Ngoc Tuyet, Voahangy Andrianaivoarimanana, Faridabano Nato, Lila Rahalison

**Affiliations:** 1 Unité Peste - Institut Pasteur de Madagascar, Antananarivo, Madagascar; 2 Département de Biologie Structurale et Chimie – Laboratoire de Production de Protéines Recombinantes et d'Anticorps - Institut Pasteur de Paris, Paris, France; 3 Laboratoire d'Entomologie Médicale – Service de Parasitologie - Institut Pasteur d'Algérie, Hamma-Alger, Algeria; 4 Département de Microbiologie - Institut Pasteur de Nha-Trang, Nha-Trang, Viet Nam; Weill Medical College of Cornell University, United States of America

## Abstract

**Background:**

Tools for plague diagnosis and surveillance are not always available and affordable in most of the countries affected by the disease. *Yersinia pestis* isolation for confirmation is time-consuming and difficult to perform under field conditions. Serologic tests like ELISA require specific equipments not always available in developing countries. In addition to the existing rapid test for antigen detection, a rapid serodiagnostic assay may be useful for plague control.

**Methods/Principal Findings:**

We developed two rapid immunochromatography-based tests for the detection of antibodies directed against F1 antigen of *Y. pestis*. The first test, SIgT, which detects total Ig (IgT) anti-F1 in several species (S) (human and reservoirs), was developed in order to have for the field use an alternative method to ELISA. The performance of the SIgT test was evaluated with samples from humans and animals for which ELISA was used to determine the presumptive diagnosis of plague. SIgT test detected anti-F1 Ig antibodies in humans with a sensitivity of 84.6% (95% CI: 0.76–0.94) and a specificity of 98% (95% CI: 0.96–1). In evaluation of samples from rodents and other small mammals, the SlgT test had a sensitivity of 87.8% (95% CI: 0.80–0.94) and a specificity of 90.3% (95% CI: 0.86–0.93). Improved performance was obtained with samples from dogs, a sentinel animal, with a sensitivity of 93% (95% CI: 0.82–1) and a specificity of 98% (95% CI: 0.95–1.01). The second test, HIgM, which detects human (H) IgM anti-F1, was developed in order to have another method for plague diagnosis. Its sensitivity was 83% (95% CI: 0.75–0.90) and its specificity about 100%.

**Conclusion/Significance:**

The SIgT test is of importance for surveillance because it can detect Ig antibodies in a range of reservoir species. The HIgM test could facilitate the diagnosis of plague during outbreaks, particularly when only a single serum sample is available.

## Author Summary

Plague is due to the bacterium *Yersinia pestis*. It is accidentally transmitted to humans by the bite of infected fleas. Currently, approximately 20 developing countries with very limited infrastructure are still affected. A plague case was defined according to clinical, epidemiological and biological features. Rapid diagnosis and surveillance of the disease are essential for its control. Indeed, the delay of treatment is often rapidly fatal for patients and outbreaks may occur. Bubo aspirate is the most appropriate specimen in case of bubonic plague, but its collection is not always feasible. The main current biological approaches for the diagnosis of human plague are F1 antigen detection, serology for antibody detection by ELISA and *Y. pestis* isolation. The biological diagnosis of plague remains a challenge because the clinical signs are not specific. In this study, we developed some simple, rapid and affordable tests able to detect specific plague antibodies. These tests can be used as alternative methods for plague diagnosis in the field and for plague surveillance.

## Introduction

Plague, a bacterial infection caused by *Yersinia pestis*, is essentially a zoonosis of small mammals such as rodents. It is occasionally transmitted to man by the bite of an infective flea [1]. The bubonic form in humans can evolve rapidly to pneumonic form if not treated early. Plague is an acute, often fatal, and potentially epidemic disease. Accordingly, plague is classified as a class I notifiable disease, subject to International Health Regulations [2]. Plague remains a serious problem for international public health. Small outbreaks of plague continue to occur throughout the world, and at least 2000 cases of plague are reported annually [3]. The disease has displayed recrudescence and geographical extension in Madagascar [4],[5]. It reappeared in Algeria in June 2003 after an absence of almost 60 years [6], and decreased in Vietnam in 2003 [7]. No natural foci of plague have been described in Algeria [8] while in Vietnam plague persists in wild animal reservoirs and is subject to an intensified monitoring program.

Bubonic plague is the major form of the disease encountered in the modern plague outbreaks. It affects mainly rural people in developing countries, whose level of education is very low. In Madagascar, plague foci matches with the poorest rural area with the most vulnerable population [9],[10]. Re-emergence of plague is associated with low sanitary condition, waste, rats abundance and proximity to rodents [11]. In fact that plague currently affects poor and vulnerable people, development of simple and affordable test which may help and contribute to the control of the disease should be taken in consideration.

The control of plague involves diagnosis and recognition of the disease [2]. For diagnosis, plague confirmation can be done by bacteriological culture (isolation of *Y. pestis* strain), by rapid diagnostic test (RDT) for F1 antigen detection (in endemic area without other confirmatory test) or by serology (four-fold rise in anti-F1 antibody titre in paired serum samples) [12].

The isolation of *Y. pestis* from clinical sample (pus of bubo, sputum) requires a laboratory with at least level II biosafety put into place [13]. Moreover, bacteriology is time-consuming, expensive and sensitive to the presence of contaminants, to patient treatment and to delays in specimen transport.

A RDT for the detection of F1 antigen, a capsular antigen of *Y. pestis*, was recently developed and could be used on bubo aspirate, sputum, sera, urine and organs removed post mortem [14]. This test may be useful for confirming clinical diagnosis and triggering alerts. It performed particularly well with bubo aspirate when bubonic plague was suspected. The development of this RDT constitutes a major advance in plague diagnosis, particularly in countries with poor medical infrastructures [15].

The serology for the detection of anti-F1 plague antibodies plays an important role in confirming plague diagnosis. Indeed, during the last outbreak of pneumonic plague in the Democratic Republic of Congo (DRC) in 2005, plague diagnosis was confirmed by evidence of seroconversion based on the anti-F1 IgG titre in paired serum samples [16]. The ELISA method is essential for retrospective confirmation when the causal agent cannot be isolated and also when only serum is available.

When neither the most appropriate specimen nor the pair of sera were available, the plague confirmation is “compromised”. A rapid test which can be performed with a unique serum collected during the early stage of the infection would be very helpful for the biological diagnosis.

For the surveys of plague infection foci, the sero-epidemiological or sero-epizootic investigations of anti-F1 antibody prevalence in human or animal population could be achieved. The species involved in the plague cycle have been identified in Madagascar and Viet Nam, but this is not necessarily the case in other countries, such as Algeria. ELISA is an efficient tool for the serology but it is difficult to carry out in field either for diagnosis or for surveillance. It requires specific equipment, expensive consumables and specific antibodies to each species for the revelation of anti-F1 to be detected. The available ELISA for human and rodent IgG detection were respectively 91.4% and 100% sensitive and 98.5% and 100% specific [17],[18]. A rapid test for anti-F1 IgG antibody detection with a “half-dipstick” format has been described for humans and animal reservoirs. Its sensitivity was 94.3% and its specificity 89.2% [19]. This format showed only a test line. A second line, absent from this test, and usually used for attesting the validity of the strip (control line) in immunocrhromatographic assays is of great importance in result interpretations.

There is still a need to develop a simple, rapid and cost-effective test for the detection of plague-specific antibodies. First, a test which can replace the ELISA and which can be used in a large-scale in the field would be beneficial. Second, another test which can be used for biological diagnosis of plague would be very helpful.

We aimed to develop two immunochromatography-based tests (dipstick). The SIgT test is able to detect total (T) anti-F1 immunoglobulin (Ig) in different species (S) (human and animal reservoirs). It was compared with ELISA. The SIgT test could have major applications in epidemiological investigations of plague and for surveillance. The HIgM test is able to detect human (H) anti-F1 IgM. Performance of the test with sera from individual with known status of plague was determined.

## Materials and Methods

### Development of the rapid serodiagnostic test

We developed two rapid tests based on a one-step, vertical-flow immunochromatography. The test consisted of a reaction pad, a conjugate pad and the absorbent pads. The reaction pad consists of a nitrocellulose membrane, with a pore size of 5 µm (Whatman International, Chateau Giron - France). The BioDot machine (BioDot, Irvine, California) was used to spray a line of Protein A (Pharmacia, Sweden) at a concentration of 2.5 mg/ml to capture Ig for SIgT test or a line of anti-human IgM (µ) at a concentration of 2 mg/ml (P.A.R.I.S, Compiègne – France) to capture human IgM for HIgM test. Protein A possesses two distinct Ig-binding domains, one that binds the Fc region of IgG and the other that binds the Fab region of Ig [20]. A second line of monoclonal anti-F1 antibody (MAbB18-1) was also sprayed on the upper part of the nitrocellulose. This Anti-F1 antibody reacts with the gold particles-F1 antigen conjugate and form a second reaction (control line), indicating that the test has been correctly performed. The control line attests the validity of the test and should always appear.

The conjugate pad consists of a polyester Accuflow P (Whatman Schleicher & Schuell, England). The colloidal gold particles (40 nm diameter) were conjugated to F1 antigen by the British Biocell International (British Biocell International Cardiff, UK). The polyester was soaked in F1 conjugated at OD = 3 and was overnight lyophilised.

The absorbent pads (sample and wicking pads) consist of cellulose filter paper (Schleicher and Schuell, Ecquevelly - France). The sample pad was soaked in blocking buffer (0.1 M sodium borate supplemented with 1% Triton X-100) and overnight dried. The wicking pad was not processed prior to use.

**Figure 1:**
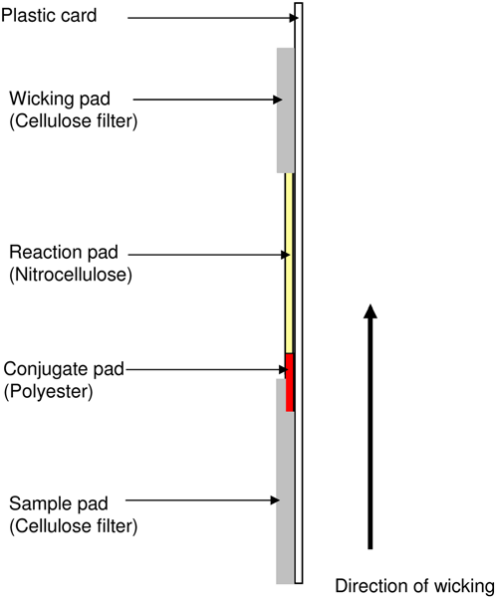
Different components of the dipstick.

A membrane-based immunochromatographic card was prepared by fixing the different pads onto a plastic card with double-sided sticky tape (Adhesives Research Ltd, Melville House United Kingdom): the reaction pad in the middle, the conjugate pad under the sample pad at the bottom and the wicking pad at the top of the card (Figure 1). The dipstick were trimmed to a width of 5 mm with the BioDot cutting machine and stored in a waterproof bag at 4°C.

**Figure 2:**
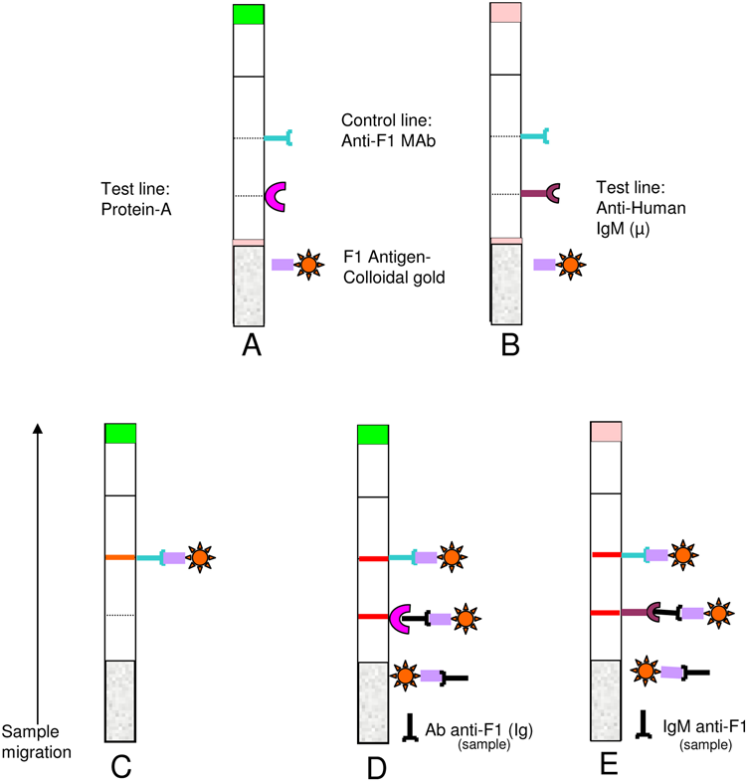
Principle of the dipstick test. A: SIgT test, B: HIgM test, C: Negative test, D: Positive test for Ig anti-F1, E: Positive test for IgM anti-F1.

The dipstick test was performed on sera or seropads (blood on blotting paper). Sera were previously diluted ten times in distilled water. For seropads, one pastille was soaked in 200 µl of distilled water and incubated for one hour at 37°C or at room temperature. This step allows the elution of the serum from the filter. The test was carried out in a plastic tube containing 150 µl of diluted sample. Diluted serum samples containing antibody absorbed from the bottom of the dipstick bound to the conjugated antigen, and the antigen-antibody binding formed complexes moved by capillary action into the nitrocellulose membrane. The complexes reacted with the immobilised protein A, generating a signal. Excess of the conjugate moved on the nitrocellulose and bound to the anti-F1 Mab, giving a second signal corresponding to the control line (Figure 2). Results were read after 15 minutes. Two pink lines appeared in positive sample and a single pink line in negative one. If the control line is absent, the test is invalid.

The shelf life of the strip for long-term storage was assessed by testing serial dilution of the positive control sera with the dipsticks stored at 60°C twice per week for three weeks. Storage at 60°C for three weeks is equivalent of two years at room temperature [21]. The detection limit of the tests was obtained by testing in duplicate serial dilutions of the positive control. For SIgT test, it was expressed as the lowest concentration of MAb anti-F1 G6-18 giving positive result. For HIgM test, the detection limit was expressed as the highest titre of sera with positive result.

### Evaluation of the plague serodiagnostic tests

The tests were evaluated on human and canine stored sera at Institut Pasteur de Madagascar and on small mammal samples freshly collected by the Pasteur Institutes of Algeria, Madagascar and Nha-Trang or from their frozen collection. The human samples tested were anonymous frozen sera from the collection of Institut Pasteur of Madagascar. Samples without turbidity, with sufficient quantity and with clearly identification number were selected and blind tests were performed. A total of 288 sera from the following groups were included in the study:

#### Sera from suspected human plague

Human samples from plague suspected cases (n = 131) were taken from sera collected during sero-epidemiological investigation in 1999 at CHU Androva-Mahajanga, the only coast plague focus in Madagascar and in 2002 at Ankazobe, one of the active foci in Antananarivo province. These sera were sampled within the framework of the routine plague diagnosis during outbreaks. Suspect plague was defined as compatible clinical and epidemiological features [2].

#### Negative control sera

Frozen sera collected from individuals in plague-free area were used as negative controls (n = 71): 24 sera from Fort-Dauphin in Madagascar collected in 2000 during malaria surveillance, 23 sera from Comoros collected in 1999 during cholera outbreak and 24 sera from Seychelles collected in 1997 during arbovirosis surveillance.

#### Sera from patient with others infectious diseases

To assess the cross-reaction with other bacterial or parasitic infections prevalent in Madagascar, samples (n = 86) collected from patients with schistosomiasis, cysticercosis, toxoplasmosis, hepatitis B, hepatitis C, streptococcosis and with unknown diseases were tested. These sera were sampled within the framework of the evaluation of a serodiagnostic test in 1997.

Evaluation on specimens from dogs (n = 63) and from rodent or small-mammals (n = 352) specimens was performed by testing sera or seropads from animals in plague foci or in areas non endemic for plague in Madagascar, Viet Nam and Algeria. Canine samples were collected in 1999 within the framework of diagnosis tool development, while rodent samples were collected during plague monitoring in rodent populations. For small mammal sera collection, we adhered to our institutional guidelines for animal husbandry and experiments.

For HIgM test evaluation, sensitivity was studied with 23 acute-phase sera specimen from bacteriologically-confirmed plague cases. Only the 26 negative controls from Madagascar and the 86 sera from patients with others infectious diseases were used for studying the specificity of this HIgM test.

### Reference tests

For SIgT test, as the purpose of this test is to be an alternative method to the ELISA in the field, ELISA was used as reference methods. Both methods were performed with each sample. Human sera were tested by human IgG anti-F1 ELISA. The sensitivity of this ELISA was 91.4%, and its specificity was 98.5% [17]. Small mammals and dogs sera were tested according to a previously described ELISA [18]. For rodent sera, revelation used a goat anti-IgG (H+L) rat Horse Radish-Peroxidase H.R.P (Sigma-Aldrich, USA) and for insectivore and dog samples, a peroxydase conjugated protein A. Samples were considered positive when the absorbance was above the defined threshold.

ELISA for rodent and insectivore was standardized by testing sera from experimentally infected animals and from animals caught in plague-free areas. For rodent ELISA, its' sensitivity and specificity were 100% [18]. For dogs, sera collected from animals in plague-endemic and plague-free areas were used to standardize the ELISA. The specificity of a positive ELISA-anti-F1 for this species was confirmed by F1 antigen-inhibition. Dog ELISA had a specificity of 97.3%. Sensitivity wasn't determined because dogs' positive controls were not available.

For HIgM test, the evaluation was carried out with sera from individual of known plague status according to the biological diagnosis by bacteriology (culture) [13]. The purpose was to have another method for plague diagnosis when bubo aspiration or sputum for the culture or the pair of sera for the serology are not available. We compare the results of HIgM test with plague status.

### Data analysis

The data, such identification number of the sample and the results of the two compared tests, were entered in Microsoft Excel sheet and analysed by Pivot Table reports. Sensitivity (Se) and specificity (Sp) estimates are given as percentages with 95% confidence interval.

### Ethical consideration

The use of the human sera from the collection of Institut Pasteur de Madagascar, including the sera from plague patients, the sera from individual in plague–free area and the sera from patients affected by others diseases, was approved by the ethics committee of the Madagascar Ministry of Health.

## Results

SIgT test was able to detect total anti-F1 antibodies in both humans and animal reservoirs species. The lower detection limit of SIgT test was 20 ng/ml of anti-F1 MAb (G6-18). The shelf-life study indicated that SIgT test was stable after storage for 14 days at 60°C, and its threshold increased to 40 ng/ml after 21 days at 60°C. The application of SIgT test to serum samples from several species was summarized in Table 1. HIgM test was able to detect human anti-F1 IgM in an unique serum. With the positive control sera, the highest IgM titre detected by this test was 1/10,000. The shelf-life study of HIgM test indicated that its performance was unaffected by storage at 60°C for 21 days. During the evaluation process, no invalid tests i.e. no tests lacked the control line were observed.

**Table 1:** Comparison of SIgT test and ELISA for human, small mammal and dog serum samples.

SIgT test	ELISA					
	Human		Small mammal		Dog	
	N (n = 223)	P (n = 65)	N (n = 278)	P (n = 74)	N (n = 49)	P (n = 14)
**N**	219 (98.2%)	10 (15.4%)	251 (90.3%)	9 (12.2%)	48 (98%)	1 (7.2%)
**P**	4 (1.8%)	55 (84.6%)	27 (9.7%)	65 (87.8%)	1 (2%)	13 (92.8%)

N : Negative, P: Positive, n: sample number.

### Application of SIgT test to human sera

**Figure 3:**
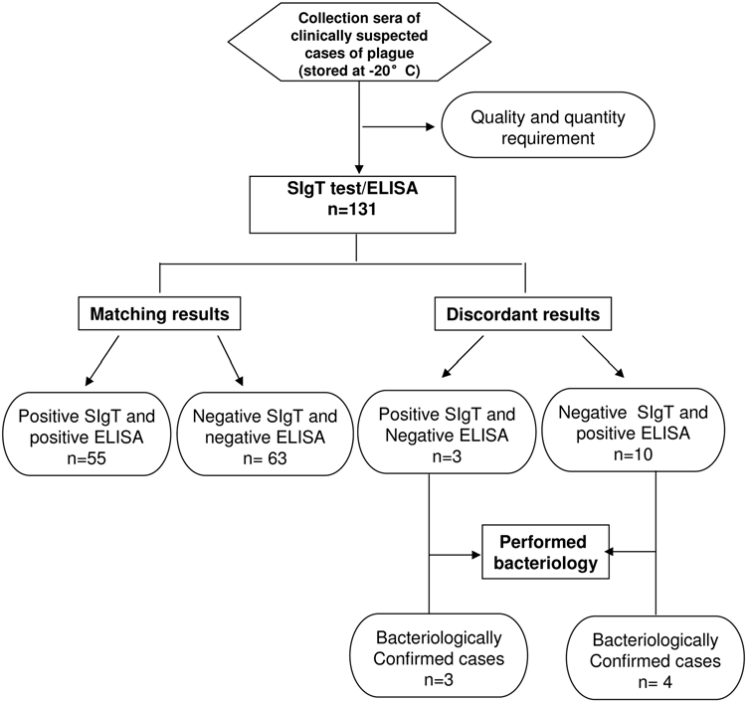
STARD flow diagram for the evaluation of SIgT test on plague suspected cases.

We tested a total of 131 sera from human clinically suspected plague from which 65 were positive by ELISA. Compared to ELISA, the SIgT test gave 55/65 (84.6%) positive results. Among ten SIgT-negative and ELISA-positive samples, six had a low IgG titre by ELISA and four were sera collected early. Among the 66 negative sera by ELISA, 63 were also negative with SIgT test and 3 were positive (Figure 3). Among the 71 negative controls tested; 70 were negative by SIgT test. The specificity of SIgT test was assessed by testing 86 samples from patients with other infectious diseases prevalent in Madagascar: 19 patients with schistosomiasis, 10 patients with cysticercosis, 14 patients with toxoplasmosis, 10 patients with hepatitis B, 10 patients with hepatitis C, 10 patients with streptococcosis and 13 patients with unknown diseases. All these sera were negative. The SIgT test performed on human samples and compared to ELISA had a sensitivity of 84.6% with 95% CI between 0.76 and 0.94 and a specificity of 98% with 95% CI between 0.96 and 1. It had a positive predictive value (PPV) of 93.2% and a negative predictive value (NPV) of 95.6% with plague prevalence estimated to 0.6% under endemic situations in Madagascar highlands.

### Application of SIgT test to small mammals sera

SIgT test was evaluated with 352 sera from animal reservoirs of plague: 88 small mammals (*Rattus rattus*, *Rattus norvegicus*) from Madagascar, 134 from Viet Nam (genera *Rattus*, *Mus*, *Suncus*) and 130 from Algeria (genera *Lemniscomys*, *Psamommys*, *Atelerix*). When compared to ELISA, SIgT test had a sensitivity of 87.8% with 95% CI between 0.80 and 0.94 and a specificity of 90.3% with 95% CI between 0.86 and 0.93. It had a PPV of 70.6% and a NPV of 96.5% with rodent plague prevalence estimated to 10.5 in context of highland endemic foci.

### Application of SIgT test to canine sera

Dogs respond to *Y. pestis* infection by producing specific antibodies against the F1 antigen of *Y. pestis*. Sixty three samples from endemic (14 sera) and from non-endemic (49 sera) areas of Madagascar were tested. Compared to ELISA, SIgT test had a sensitivity of 93% with 95% CI between 0.82 and 1.0 and a specificity of 98% with 95% CI between 0.95 and 1. It had a PPV of 92.8% and a NPV of 98% with a plague prevalence estimated to 23.8% under plague endemic area situation.

### Evaluation of HIgM test

We evaluated HIgM test with 23 acute-phase sera from bacteriologically-confirmed plague patient and 110 plague-negative (24 negative control from Madagascar and 86 with others infectious disease). All these sera were from the collection of Institut Pasteur de Madagascar. Results of HIgM tests are presented according to the patient status for plague (Table 2). HIgM test gave 19/23 positive results. Among the 4 discordant results (HIgM test negative but plague status confirmed by bacteriology), 2 samples were collected within three days after the disease onset. HIgM test was found to have a sensitivity of 83% with 95% CI between 0.75 and 0.90 and a specificity of 100%. This test had a PPV of 100% and a NPV of 96.49% with plague prevalence estimated to 0.6% stated above. No cross-reactivity with HIgM test was detected within the 86 sera from patient infected with other diseases.

**Table 2:** Use of HIgM test for plague diagnosis.

HIgM test	Patients status	
	Plague negative (n = 110)	Plague confirmed (n = 23)
**N**	110 (100%)	4 (17%)
**P**	0 (0%)	19 (83%)

## Discussion

We developed and evaluated two tests for the qualitative detection of plague anti-F1 antibodies in sera: the SIgT test for total Ig anti-F1 antibodies during and after plague infection in humans, rodents and other animals and the HIgM test for anti-F1 IgM in humans.

Using ELISA as reference method, SIgT test detected plague antibodies in human with a sensitivity of 84.6% and a specificity of 98% according to the reference test ELISA which sensitivity and specificity were respectively 91.4% and 98.5% [17]. By this comparison, 3 discordant results SIgT positive-ELISA negative were obtained with sera collected from bacteriologically-confirmed plague cases. Of the 10 samples SIgT test negative-ELISA positive, four sera were from bacteriologically-confirmed plague cases (Figure 3). These are SIgT test false negative. The limitation of this new test is about the low positive sample with ELISA that could be negative by SIgT test. Although less sensitive (but specific), this new test would be useful in case of plague outbreak since it could give rapid information on the human plague situation in a studied area. It is also particularly interesting for retrospective investigation when only serum is available.

In addition, since SIgT test detects plague-specific antibodies in many species of animal reservoirs, it is suitable for large scale serological survey of reservoirs in remote and impoverished areas endemic for plague. This could help to determine the risk of plague in a given zone, leading to a progress in disease prevention. SIgT test, used for canine sera proved sensitive and specific enough for this purpose, since it provided evidence of plague antibodies production in 93% of the samples collected from area of endemic plague, whereas over 98% of the samples from areas considered free of plague tested negative. Indeed, dogs are useful sentinels of plague prevalence, since animals living in or in adjacent to areas endemic for plague may be in contact with *Y. pestis* by infected flea bites or by consuming infected prey. They may develop high antibody titre without plague symptoms [22]. Moreover it is easier to manage dogs than small mammals' surveillance whose study is tedious (number of samples to be collected and analysed).

HIgM test was developed for the detection of anti-F1 IgM in humans to provide an alternative diagnostic method for plague, particularly when serum is the only clinical specimen available. HIgM test in plague confirmed cases gave a specificity of 100% and a sensitivity of 83%. This low sensitivity will generate some false negative results. However, of the 4 “false negative” samples; 2 were taken early (within 2 days after onset of the disease) before IgM was likely to be detectable in blood and 2 were collected 1 week after the onset of the disease. Owing to its high specificity, HIgM test could be used with significant advantage on serum samples collected during the acute phase as early as three days after onset of the disease. It could be performed with only a single serum sample while plague diagnosis by ELISA usually need a pair of sera (early and late sera collected at 4–6 weeks interval) [2].

Our aim was to develop some simple, rapid and affordable tools for a large scale use in plague monitoring (seroepidemiological investigations) and as an alternative test to ELISA.

In the majority of endemic area, particularly in Madagascar, the poor sanitary and economic situation renders difficult the control and surveillance of plague. Bacteriology techniques including culture-isolation and mouse infection require appropriate laboratory. In developing countries, at the district level, simple tests like the dipstick assay can be implemented in the health centres. Most of the suspected cases of plague are detected in remote villages in rural area. As soon as transport of specimen from these places to a central laboratory is long and difficult, it is essential to possess an alternative tool for plague diagnosis and surveillance on site.

A pilot assessment of the new tests by users at the periphery level could be considered to define the utility and performance of these tools in field conditions.

In conclusion, the rapid serodiagnostic tests developed in this study are promising, not only for epidemiological studies, but also for the surveillance of reservoirs and active foci and for plague diagnosis. Application in case of bioterrorism attack can also be considered as *Y. pestis* is recognized as biological weapon [23].

## Supporting Information

Alternative Language Abstract S1 Translation of the Abstract into French by Lila Rahalison
(0.03 MB DOC)

Checklist S1 CONSORT Checklist
(0.08 MB PDF)
