# Supplementary material for: Development and Evaluation of Two Simple, Rapid Immunochromatographic Tests for the Detection of Yersinia pestis Antibodies in Humans and Reservoirs
Source: PLoS Negl Trop Dis. 2009 Apr 28;3(4):e421. doi: 10.1371/journal.pntd.0000421 (PMC2668800; doi:10.1371/journal.pntd.0000421)
Supplement: Alternative Language Abstract S1 — Translation of the Abstract into French by Lila Rahalison (0.03 MB DOC) [file pntd.0000421.s001.doc]

**RESUME
Introduction**

Les Outils de diagnostic et de surveillance de la peste ne sont pas toujours disponibles dans la plupart des pays touchés par la maladie. La confirmation biologique par isolement de *Yersinia pestis* est longue et difficile à effectuer dans les conditions de terrain. Les tests sérologiques tels que l’ELISA nécessitent des équipements spécifiques qui ne sont pas toujours disponibles dans les pays en développement. Outre les tests rapides de détection de l'antigène, un test de sérodiagnostic rapide pourrait être utile pour contrôler la peste.

**Méthodes / Principaux résultats**

Nous avons développé deux tests rapides immunochromatographiques de détection d’anticorps dirigés contre l'antigène F1 de *Yersinia pestis*. Le premier test, SIgT, qui détecte les Ig anti-F1 (Ig) totaux (T) chez plusieurs espèces (S) (homme et réservoirs animaux), a été développé afin de disposer sur le terrain d'une méthode alternative à l’ELISA. La performance de ce test est évaluée en comparaison avec le test ELISA sur des échantillons provenant de patients et des animaux. Utilisé sur des sérums humains, le test SIgT montre une sensibilité de 84,6% (IC 95%: 0.76-0.94) et une spécificité de 98% (IC 95%: 0.96-1). Evalué sur des sérums de rongeurs et autres petits mammifères, il montre une sensibilité de 87,8% (IC 95%: 0.80-0.94) et une spécificité de 90,3% (IC 95%: 0.86-0.93). Sur des échantillons provenant de chiens, animaux sentinelles, la performance du test est améliorée. En effet, une sensibilité de 93% (IC 95%: 0.82-1) et une spécificité de 98% (IC 95%: 0.95-1.01) sont obtenues. Le deuxième test HIgM qui détecte les IgM anti-F1 chez l'homme (H) est développé afin de disposer d’une autre méthode pour le diagnostic de la peste. La sensibilité de ce test est de 83% (IC 95%: 0.75-0.90) et sa spécificité à 100%.

**Conclusion**

Le test SIgT test est d'une importance capitale pour la surveillance, car il peut détecter les anticorps dans une gamme d'espèces de réservoirs. Le test HIgM pourrait faciliter le diagnostic de la peste lors d’épidémies, en particulier lorsqu’un seul échantillon de sérum est disponible.
